# Primary retroperitoneal carcinosarcoma in a child: a case report

**DOI:** 10.1186/1477-7819-8-99

**Published:** 2010-11-18

**Authors:** Feng Xu, Yangqing Huang, Jiamei Yang

**Affiliations:** 1 Special treatment department, Eastern Hepatobiliary Surgery Hospital, 225 Changhai Road, Shanghai 200438, China

## Abstract

Carcinosarcoma is a rare biphasic malignancy consisting of intermixed epithelial and mesenchymal elements. Carcinosarcoma is particularly rare among children. We accepted a 7 year old patient with retroperitoneal carcinosarcoma. The tumor was totally resected and no recurrence is found 11 months after operation. Literatures has been reviewed and there are few reports of primary retroperitoneal carcinosarcoma in children up to date. So we report the patient's clinical character, surgical resection, pathological and immunohistochemical analysis.

## Background

Carcinosarcoma is a rare malignancy consisting of intermixed epithelial (carcinoma) and mesenchymal (sarcoma) elements, mostly arising from glands and female reproductive system [1,2]. Carcinosarcoma is particularly rare among children. There are few reports of primary retroperitoneal carcinosarcoma of children in english literatures up to date. We report our experience in treating an unusual case of retroperitoneal carcinosarcoma in a 7 year old patient, its clinical characteristics, surgical resection, pathological and immunohistochemical analysis.

## Case Presentation

**Figure 1:**
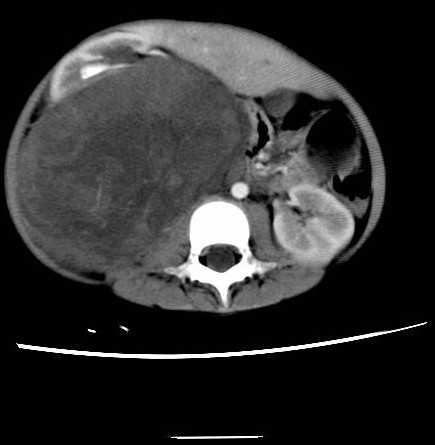
**CT scan image of the tumor**.

A 7 year old female was admitted due to two month history of epigastric pain with anorexia, who was found to have an epigastric mass. Past medical history was unremarkable. Abdominal exam revealed a distended abdomen with a palpable mass at the right upper quadrant. The mass was estimated to be around 15 cm, moderate hardness with clear edge, but not movable and no tenderness. No abdominal wall venous varicose was noticed. Bowel sounds were present and normal. Lower extremity edema was absent, bilaterally. Laboratory test showed normal hepatic and renal function. AFP 4.9 μg/L, CEA 0.6 μg/L, CA 19-9 11.7 U/ml. Ultrasound exam showed a large well-circumscribed solid mass with irregular internal echo signals located in the right upper quadrant extending. On CT, the right upper quadrant solid mass was measured at 20 cm longitudinally. Tumor image was mildly enhanced at artery phase, which rapidly phased out at venous phase and delayed phase. The adjacent kidney, liver, stomach, duodenum, transverse colon, abdominal aorta and vena cava were all compressed or distorted (Figure 1). No tumor was found in chest, other parts of abdomen and pelvis cavity by CT scan.

### Surgery report

**Figure 2:**
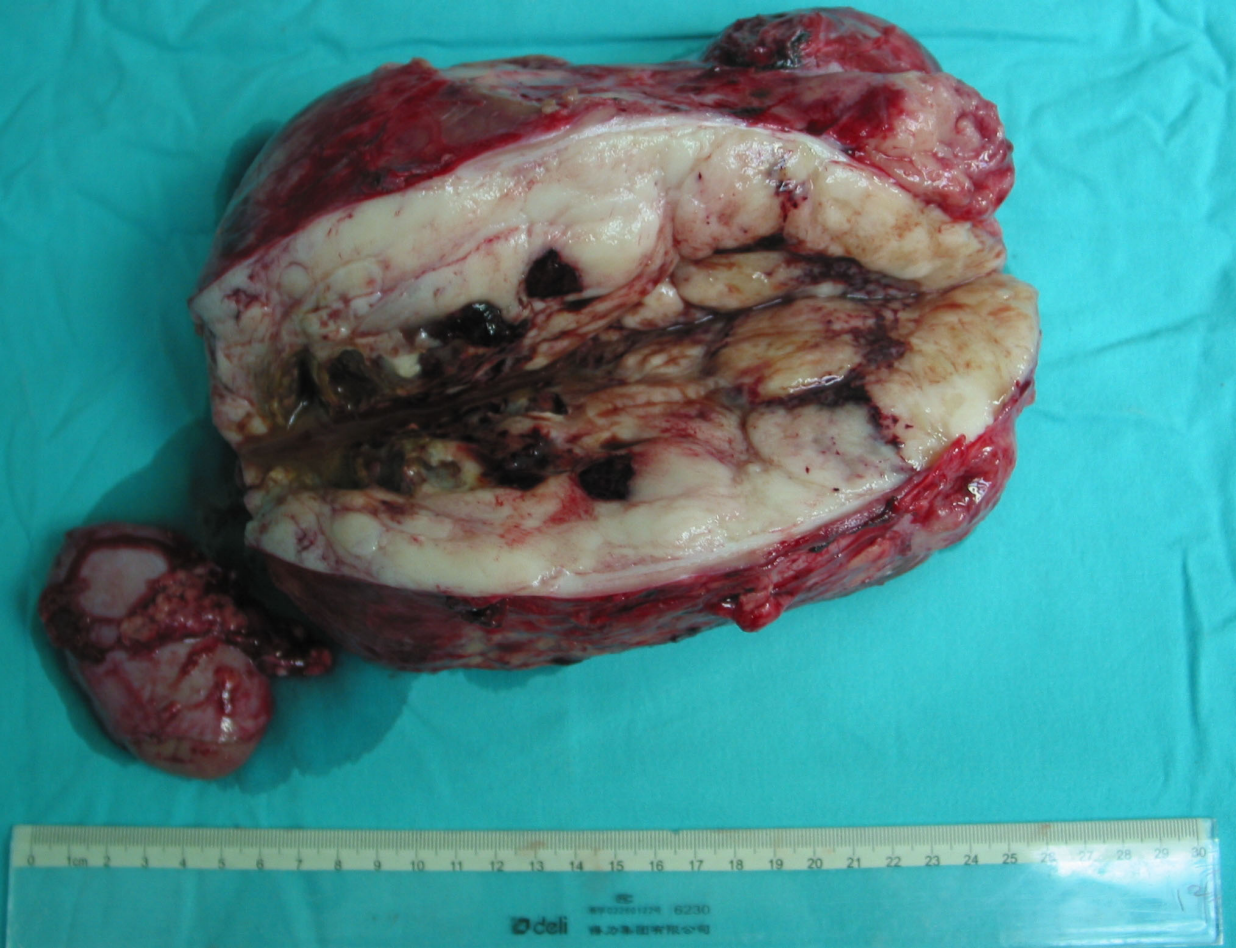
**The tumor measured at 21 × 14.6 × 12.1 cm with intact capsule**. The removed right kidney was next to the tumor. Section of the tumor revealed greyish-white color, "fish-meat" shape inside with part of the capsule. There were spotted bleeding and cystic necrosis cavities filled with brown color fluid ranging 1.2 - 3.0 cm in diameter.

Preoperative diagnosis was retroperitoneal mass with unknown origin. Standard upper gastric laparotomy was performed under general anesthesia. Intraoperative findings: the entire right upper abdominal cavity was occupied by a large solid tumor with faint purple color and moderate hardness. The adjacent right kidney was dislocated inferiorly forming an elongated kidney pedicle. The liver (including portal structure) was pushed leftward and anteriorly, right hepatic lobe appeared thinner. However, the liver was soft, no mass, cirrhosis change or other abnormalities were appreciated. The adrenal gland, duodenum, transverse colon, abdominal aorta and vena cava were all compressed by the tumor, but can be dissected from the tumor easily and no tumor invasion was detected among these adjacent organs. The reproductive system organs were free from the tumor compression. There was a tight adhesion between the tumor and the right kidney, making it hard to judge whether the tumor originated from the kidney or the tumor infiltrated the kidney. Frozen section of the kidney sample reported "malignant tumor without identifiable kidney glomerulus structure, possible tumor infiltration". Complete retroperitoneal tumor resection plus right nephrectomy was thus performed. Macroscopically, the tumor weighed 3.5 Kg, measured at 21 × 14.6 × 12.1 cm, encapsulated with fibrous tissue. Section of the tumor revealed "fish-meat" like greyish-white color inside with spotted bleeding and cystic necrosis cavities ranging 1.2 - 3.0 cm in diameter filled with brown color fluid (Figure 2). Right kidney section did not showed obvious mass.

### Pathological analysis

**Figure 3:**
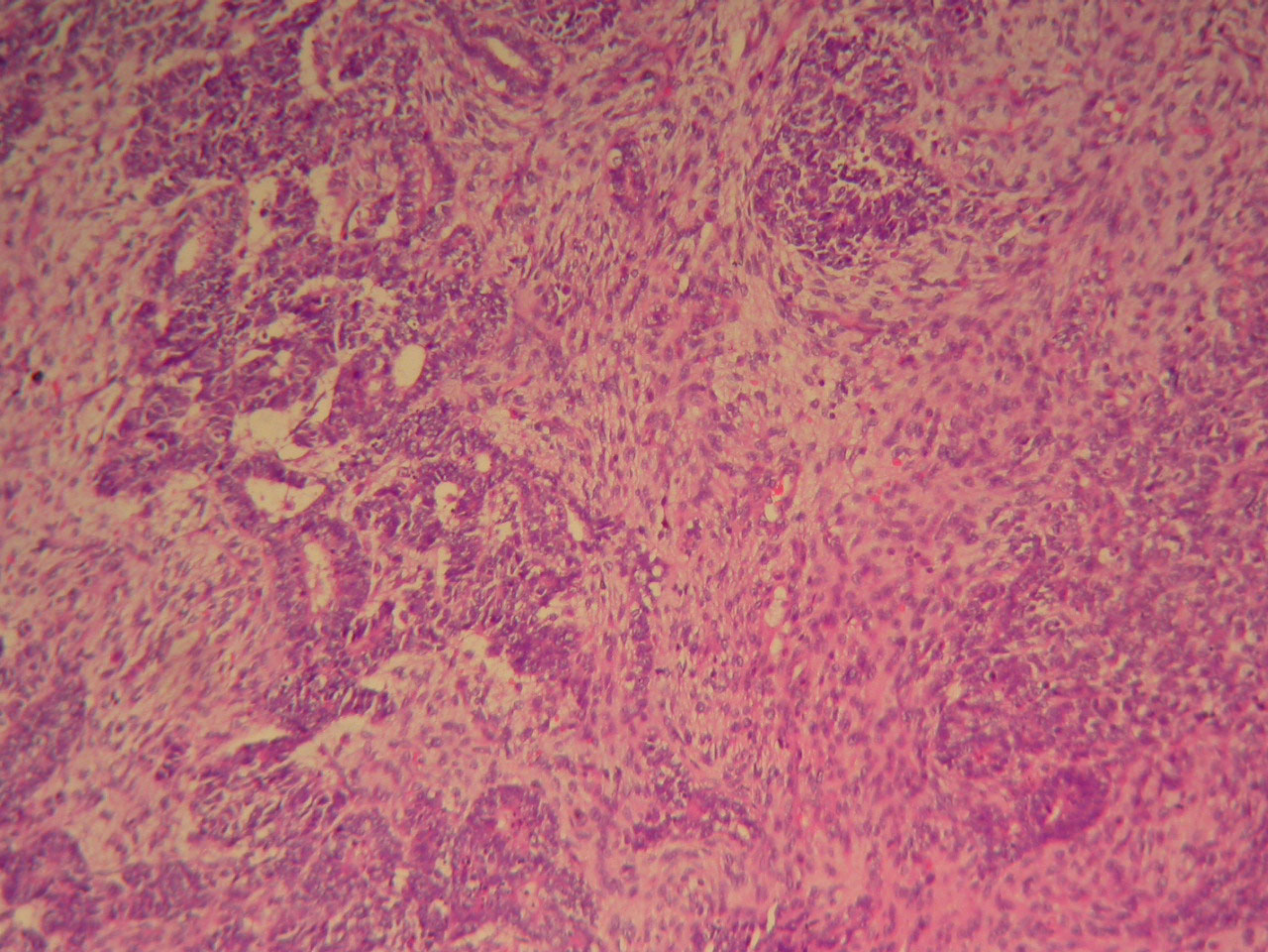
**HE staining image of the tumor tissue**. HE staining showed that the tumor tissue consisted of two well distinguished types of cells, large spindle cells lining in net with identifiable nuclear mitosis and epithelial nest or adenocarcinoma cells lining around microvascular structure.

**Figure 4:**
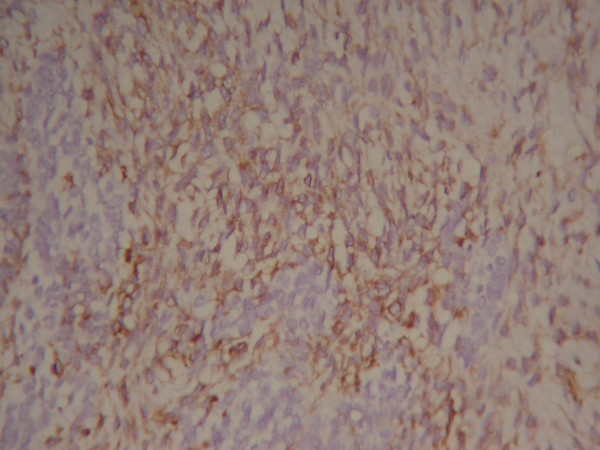
**Histological study revealed Vimentin stain (+)**.

**Figure 5:**
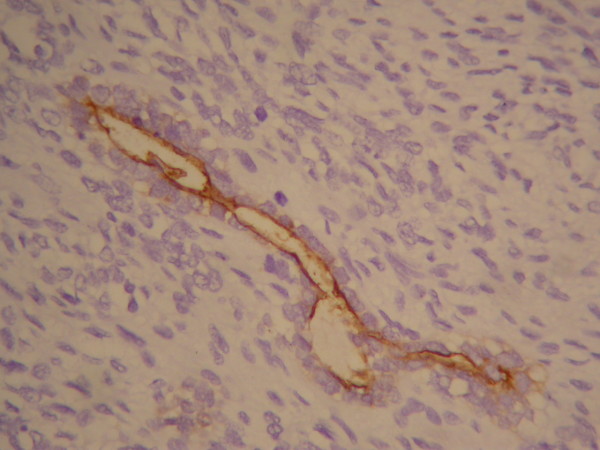
**Histological study revealed EMA (+)**.

**Figure 6:**
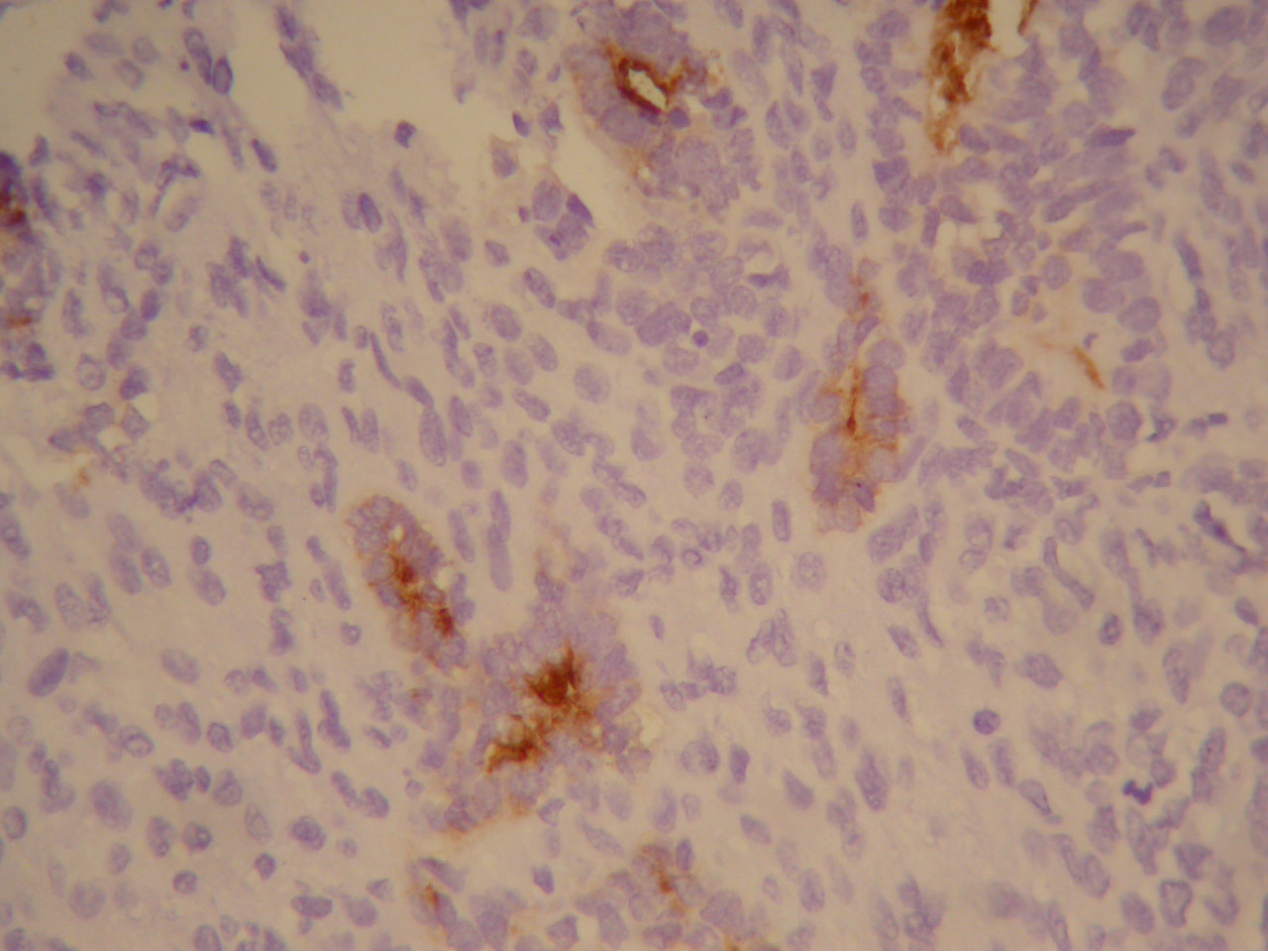
**Histological study revealed Cytokeratin stain (+)**.

Haematoxylon-Eosin (HE) staining showed that the tumor tissue consisted of two well distinguished types of cells, large spindle cells lining in net with identifiable nuclear mitosis and epithelial nest or adenocarcinoma cells lining around microvascular structure (Figure 3). Large area of bleeding and necrosis were present inside the tumor with renal cortical infiltration. There was no infiltration into the renal medullary tissue. Surgical margin was negative for tumor cells. Immunohistochemical analysis revealed Vimentin stain (+), Cytokeratin stain (+), EMA (+) (Figure 4, 5, 6). Pathological diagnosis: Retroperitoneal carcinosarcoma with right renal cortical infiltration.

The patient had an uneventful recovery following the surgery and was discharged home on postoperative day 7. On 11 months follow up, the patient did well and gained weight. She was symptom free with normal renal and hepatic function. There was no tumor recurrence was found on CT scan

## Discussion

Primary tumors arising from retroperitoneal space are rare, most are soft tissue tumors. The incidence of retroperitoneal malignancies (mainly sarcoma, such as leiomyosarcoma, fibrosarcoma) is reported as 2.7/10^6 and did not change significantly over the years [3,4]. Carcinosarcoma is very rare, usually occurs in gland tissue and female reproductive system. However, there is no report of primary retroperitoneal carcinosarcoma so far, especially among children.

Carcinosarcoma consists of mixed epithelial carcinoma and mesenchymal sarcoma components. The ratio of these two components may vary, however, they are related and share many same biological characters [5]. There have been many hypotheses regarding the origin of carcinosarcoma with main dispute on whether the two components of the tumor are homogeneous. Meryer [6] first classified carcinosarcoma into three types. Type I Collision tumor: the two related carcinoma and sarcoma happen to occur and fuse at the common boarder and infiltrate each other to form a single tumor. Type II Combination tumor: The two type of cell lines in carcinosarcoma derive from the same pleuripotent stem cells to form a single tumor. Type III Composition tumor, in which both components derive from the same tissue concomitantly. Peterson's study [7] supported that the carcinosarcoma was developed from one same non-specified stem cell line proliferating along both epithelial and metodermal cell lines. Eventually these two related cells formed a single tumor. Recently, most studies agree that carcinosarcoma origins from monoclonal stem cells, rather than multiclonal stem cells [8].

Carcinosarcoma has no specific clinical symptoms or signs. There is also no specific laboratory and imaging study reported. It is difficult to differ it from other retroperitoneal malignancies before surgical resection. The diagnosis of carcinosarcoma is thus mainly by pathological and immunohistochemical analysis. Most primary retroperitoneal tumors are found due to abdominal pain or abdominal mass. Ultrasound and CT imaging studies can help delineate tumor location and anatomy in relation to adjacent organs in order to make a treatment plan. Final diagnosis is usually immunohistological. H & E staining can clearly show characteristic epithelial and mesenchymal cell components and their differentiation degree. Immunohistological study can determine the tumor components by specific labeling techniques. Most used labeling techniques include vimentin, VI, CD99 for mesenchymal tissue and epithelial membrane antigen (EMA) and cytokeratins for epithelial tissue [5,9,10]. Our patient presented with abdominal pain and retroperitoneal mass with unknown origin. Final diagnosis of carcinosarcoma was confirmed by H & E staining and immunohistochemical analysis showing Vimentin (+), Cytokeratins (+) and EMA (+). This also excluded sarcomatoid carcinoma. Sarcomatoid carcinoma is one kind of carcinoma which comes from epithelial cell. The sarcoma-like tissue differentiates from epithelial cell with expression of mesenchymal markers. So is not a true sarcoma or carcinosarcoma [10].

Carcinosarcoma is a highly malignant and invasive tumor. Retroperitonal carcinosarcoma can often occur insidiously without obvious signs or symptoms until the tumor grow large enough to compress adjacent organs. Currently, surgical resection is still the main and most effective treatment method [11,5]. Surgery requires complete resection of the tumor together with infiltrated adjacent organ tissue. It has been reported that the prognosis depended on the negative surgical margin [12,13]. Adjuvant chemotherapy may not be satisfactory in some patients, but many studies showed that combined surgery and chemotherapy or radiotherapy can often achieve a much better prognosis, especially among children. This is due to: 1. Retroperitoneal tumor is usually very large upon discovery with many complex adjacent organ tissue infiltration making complete surgical resection a challenge. So it is not uncommon that residual tumor or positive surgical margin occur more often than tumors of other locations. Mendenhall [14] summarized several large patient population studies and found that only 50-67% of retroperitoneal sarcoma achieved complete surgical resection. Thus, combined chemotherapy or radiotherapy are often used to compensate and hope to reduce postoperative tumor reoccurrence [15,16]. 2. In contrast with adult, sarcoma in children are mainly composed of striated muscle sarcoma and fibrosarcoma, which are usually sensitive to chemotherapy and radiotherapy. So chemotherapy and radiotherapy often improved survival rate of sarcoma in children [4]. In our case, the tumor and the involved right kidney were completely removed with negative surgical margin. With only one kidney remaining in a 7 year old child, we did not employ chemotherapy or radiotherapy postoperatively so as to avoid possible severe complications. We follow the patient closely, she is doing very well 11 months after the surgery without tumor recurrence was found.

Currently, there has been no prognosis study on primary retroperitoneal carcinosarcoma. However, inferring from prognosis study of carcinosarcoma in other locations, prognosis of retroperitoneal carcinosarcoma can be very poor, given its highly malignant, rapid aggressive infiltration and easy postoperative metastatic nature [11,17].

## Conclusions

Carcinosarcoma is a rare biphasic tumor with high malignancy, aggressive invasiveness and poor prognosis. Preoperative diagnosis is often difficult, especially for retroperitoneal carcinosarcoma, due to its insidious onset and lack of specific laboratory and imaging study available. Confirming diagnosis requires immunohistological studies. Surgical resection is still the most important treatment, plus adjuvant chemotherapy and possible targeted therapy could improve survival rate. However, satisfactory result is still hard to achieve. This is especially the case among children due to the severe complication from chemotherapy limiting it application in these patients. The prognosis of our patient could be get depending on further follow ups.

## Consent

Written informed consent was obtained from the patient's father for publication of this case report and any accompanying images. A copy of the written consent is available for review by the Editor-in-Chief of this journal.

## Competing interests

The authors declare that they have no competing interests.

## Authors' contributions

JMY proposed the study. FX and YQH wrote the first draft. XF and YQH analyzed the data. JMY is the guarantor. All authors read and approved the final manuscript.
